# Experiences of patients with common mental disorders concerning team-based primary care and a person-centered dialogue meeting: An intervention to promote return to work

**DOI:** 10.1371/journal.pone.0271180

**Published:** 2022-07-08

**Authors:** Ausra Saxvik, Karin Törnbom, Eva-Lisa Petersson, Dominique Hange, Shabnam Nejati, Cecilia Björkelund, Irene Svenningsson

**Affiliations:** 1 Primary Health Care/Department of Public Health and Community Medicine, Institute of Medicine, Sahlgrenska Academy, University of Gothenburg, Gothenburg, Sweden; 2 Research, Education, Development & Innovation, Primary Health Care, Region Västra Götaland, Sweden; 3 Department of Social Work, University of Gothenburg, Gothenburg, Sweden; PLOS: Public Library of Science, UNITED KINGDOM

## Abstract

**Objectives:**

Common mental disorders in combination with work-related stress are widespread in the western world, not least in Sweden. Various interactive factors, primarily work-related, have impact on the return to work process, for example; a supportive communicative function between the person on sick leave and the employer may facilitate this process. The aim was to investigate experiences of being part of a collaborative care model including a person-centered dialogue meeting with the employer and with a rehabilitation coordinator as the moderator.

**Methods:**

A qualitative design based on individual interviews with 13 persons diagnosed with common mental disorders who participated in an extensive collaborative care model, called the Co-Work-Care model. Persons were recruited as a heterogeneous sample with respect to age, gender, work background, and time since the intervention. All interviews were analyzed with Systematic Text Condensation.

**Results:**

Five codes synthesized the results: 1) A feeling of being taken care of, 2) Collaboration within the team was perceived as supportive, 3) An active and sensitive listener, 4) Structure and planning in the dialogue meeting, 5) The person-centered dialogue meeting was supportive and provided increased understanding.

**Conclusions:**

Participants experienced the close collaborative contact with the care manager and the rehabilitation coordinator as highly valuable for their rehabilitation process. Participants valued a well-structured dialogue meeting that included initial planning and a thorough communication involving the patient, the employer, and coordinator. Further, participants appreciated having an active role during the meeting, also empowering the return to work process.

## Introduction

Depression, anxiety syndromes, and mental stress disorder are common in Europe and are among the most frequent reasons for sick leave in Sweden. [1,2]. Further, a majority of all out-patient physician contacts for anxiety and depression occur in primary care [3].

A recent systematic review of mental health, work, and sick leave [4] found that more than 90% of psychiatric diagnoses in new cases of sickness were of depression, anxiety or stress related. Many of these individuals have additional symptoms, both of mental and physical character, such as chronic pain, sleep disturbances, hypertension, cardiovascular or metabolic disorders, and often have an established contact with the primary care center [4].

Sick leave episodes are increasing in Sweden, more so for women than for men. The increase is primarily due to common mental disorders (CMD), and CMD are now the most common cause of sick leave in Sweden [3]. Factors that have an impact on increased sick leave are severe depression, as well as various conditions at the workplace. However, we still do not know how different factors interact on an individual level [5]. Further, the increase of patients with CMD suggests a need to improve care and treatment available for these patients.

Various factors that interact, both at work and in one’s private life, have an impact on the development of CMD, leading to sick leave. Some of the most common causes are high demands at work and private conflicts or personal crises [6]. It was previously shown that job support and experienced justice at work were protective factors that reduced the risks of emotional burnout [7]. With regard to the return-to-work (RTW) process, interventions aiming to bridge between health care and working life showed promising and positive outcomes [8]. Still, while substantial research has focused on RTW, only a small number of studies have focused on workplace-based interventions and mental health conditions [8].

The importance of collaboration among the patient, the Primary Care Center (PCC), and the workplace highlights the need for a supportive organization at the PCC to accomplish a person-centered care at different stages of the disease, while at the same time, providing support for patients in their return to work (RTW). Internationally, the type of organizational change called Collaborative Care [9], in which a care manager (CM) coordinated the care by maintaining a close and regular contact with patients, showed not only to improve symptoms of depression but also enhanced the individual’s physical health [10].

The collaborative care model with a CM has generated positive results for patients with depression in terms of a decrease in depressive symptoms [11,12]. A rather new competency in health care, a rehabilitation coordinator (RC), has been introduced in Swedish primary care, working together with the CM and the general practitioner (GP). The RC’s main function is to support patients on sick leave during their rehabilitation and to coordinate contacts with the patient´s employer during the RTW process.

In the current study, we focused on patient´s experiences of participating in a model with extensive collaborative care, i.e. the Co-Work-Care (CWC) model, which is a working method that involves the entire PCC. However, the most important roles are played by the CM, the RC, and the GP, working as a team together with the patient. The collaborative care is started as soon as the patient wants to, often within a few days after their initial contact with the PCC. An important part of the RTW process in the CWC model is the person-centered dialogue meeting between the patient and the employer, which takes place in the model (not later than 3 months from start of sickness period), where the patient can present thoughts and reflections about RTW. In this way the patient’s needs, preferences and experiences, the prerequisites for person centered care, forms the basis also for the workplace collaboration in the CWC model [12]. It is of great importance to evaluate this model of supportive and collaborative care at the PCC from a patient’s perspective.

The aim of this study was to explore experiences of persons with common mental disorders who participated in the Co-Work-Care model with a person-centered dialogue meeting and a rehabilitation coordinator as the moderator. This main scope was covered in the results section.

## Materials and methods

This study adheres to the consolidated criteria for reporting qualitative research (COREQ) guidelines. A qualitative research design with semi-structured individual interviews was performed. Interviews were analyzed with systematic text condensation (STC), which is a strategy for analysis developed to suit most of the methods for qualitative data [13]. The STC model offers a process of reflexivity and feasibility, while maintaining a responsible methodological approach. An important example of this responsible approach is that individual statements are to a great extent preserved in their original form, thereby enabling the reader to gain a better understanding of participants’ experiences [13].

### The Co-Work-Care model

In this study, experiences of the Co-Work-Care (CWC) model [12] were studied from a patient perspective. This model included an in-depth collaboration among the CM, RC, and GP, followed by a person-centered dialogue (PCD) meeting between the patient and the employer, with the RC attending as a dialogue moderator.

Initially, the patient with CMD meets their GP, who assesses the need for a sick leave certificate and refers the patient to a CM and/or a RC. The in-depth collaboration between the CM and RC is based on the unique prerequisites for each PCC. During the care process and collaboration among the CM, RC, and GP, the patient’s situation becomes clearer and forms the basis for the PCD meeting.

Commonly, the CM is involved in the patient’s situation from day one, and the RC is mostly involved in the PCD meeting and in all collaboration surrounding the patient. However, the structure of these roles varies among the PCCs. Depending on the size of the PCC and other practical circumstances, in some cases the same nurse has the role of being both the CM and the RC. In the present study, this was the case for six out of thirteen participants; hence, the CM and the RC are referred to “CM/RC” in cases when this is the same person in both roles.

#### The person-centered dialogue meeting

When forming the structure and content of the PCD meeting, a theoretical framework for a traditional convergence dialogue meeting was used [14]. The main aim of this meeting was to facilitate the patients’ RTW process [15] and thus shorten their sick leave period [14].

The convergence dialogue meeting was modified to meet the preconditions at the primary care centers and is thus referred to as the person-centered dialogue (PCD) meeting. The PCD meeting is based on the patients’ experiences of their situation, which clarifies their needs and requirements. The main aim of the meeting is to give the patients an opportunity to sit down with their employer and the RC in a calm and neutral environment (preferably at the PCC), and to describe their situation in their own words in front of the employer. The meeting is based on the patient’s experience of her/his situation and thus centered on the patient as a person. Before the meeting, the RC prepares written and oral information about the aim of the meeting in order to prepare the patient and the employer. The RC leads the PCD meeting and is responsible for ensuring that the patient’s needs and perception of the situation stays in focus. The RC starts the meeting with the following question for the patient: “Can you tell us about how you perceive your situation?” and the following question for the employer: “How do you perceive the patient’s situation?”. The meeting continues for about 45–60 minutes. The employer and the patient, together with the RC, end the meeting with a conclusion and an agreement for how to proceed [12].

### Sampling and participants

Participants were recruited from a database with patients from eight different PCCs who had been a part of the CWC model between September 2018 and August 2020. In order to gain broad information about the research questions, participants were purposively selected with varying age, gender, and from different PCCs. An initial aim was to include participants born in other countries than Sweden, but this was not possible due to a lack of patients with this background in the database. Of sixteen persons contacted, thirteen individuals, eleven women and two men agreed to participate and were sent extensive information about the study. Regarding occupation; eight participants worked in nursing professions (i.e. care assistant, nurse, child-care worker or personal assistant), one social worker, one bank-clerk, one shop assistant, one entrepreneur and one participant was studying. At time of the interview participants worked between 50–100%. Average time since intervention was 6 months.

All participants signed a written informed consent that was sent back to the research group per mail. All persons included had received a diagnosis of depression, anxiety and/or stress-related syndromes (CMD) when participating in the CWC model. All participants were of working age (28–64 years old).

### Data collection

Interviews were conducted during September and October 2020; thus, between one month and two years had elapsed since the end of the CWC intervention. Due to the COVID-19 pandemic, face-to-face interviews were not considered. Instead, participants could choose either between participating in a telephone interview or an interview conducted during a video conference call. All participants chose telephone interviews, and a few stated that due to their CMD, they were not able to sit in front of a screen for any period of time.

Participants were interviewed by phone for an average of 45 minutes. Prior to the meeting, participants were asked if they preferred an individual phone call or if they wanted both of the main authors to be present during the interview. Thus, interviews were carried out either by KT or AS, or by both of the authors in a three-way phone call. Interview questions were open-ended and focused on areas relevant to the research aims, i.e. experiences of the relationships with and support/help from the CM, RC, and the GP, as well as experiences of the person-centered dialogue (PCD) meeting (structure, content, purpose, overall feelings and the role of the RC).

Supplementary questions were posed to further probe participants’ answers, thereby yielding an interview material that was rich in content. Both interviewers encouraged participants to speak freely within the frame of the subject, allowing additional thoughts to emerge. The interview guide in Swedish and English; see **S1 File.** and **S2 File**. All data were transcribed verbatim. Although a few interviews had low sound levels, all transcriptions were possible to carry out.

### Analysis

The analysis was conducted with the STC according to Malterud [13] and was a collaboration among KT, AS, ELP, IS, CB, and DH. KT is a social worker with a doctoral degree and experience from psychotherapy in outpatient care. AS is a general practitioner and a doctoral student. ELP is an occupational therapist, and IS is a district nurse; both are associate professors with long experience from primary health care. CB is a general practitioner and professor, and DH is a general practitioner and associate professor. Throughout all steps in the analysis, discussions between researchers with different backgrounds were used to obtain a deeper and more nuanced understanding of the material. Five of the researchers conducted the analyses and all were females.

Malterud [13] does not use the concept of saturation concerning sampling methodology. Instead, it is important to provide coherent stories to establish an adequate information-rich sample grounded in empirical data. Thus, the analysis was data-driven, with no theoretical framework as model.

Procedures for analyzing according to the STC [13] were followed and included the following steps: 1) To establish an overview of data, all interviews were read several times by the authors AS and KT, to create a broader analytic space. 2) Various aspects of the participants’ experiences of the CWC model and their relation to the CM, RC, and GP were identified and labeled as different meaning units. In the same manner, participants’ experiences of the PCD meeting were identified and coded in meaning units. 3) In this step the codes were abstracted independently by the authors AS and KT–from code to meaning. Codes that were related formed code groups, that were successively condensed and revised through discussions between all authors. When forming condensed code groups, the original terminology used by participants was kept, making sure to represent all participants who provided information in this specific area. 4) Finally, the contents of the condensed code groups were synthesized. This step mainly concerns making sure that a trustworthy story, i.e. one that answers the study questions in a satisfactory manner, is being produced [13].

### Ethical statement

Ethical approval was obtained from the Regional Committee for Medical Research Ethics in Gothenburg (Dnr 459–17) and from the Swedish Ethical Review Authority (Dnr 2020–02125). All participants received verbal and written information about the study before deciding on participation. All participants signed a written consent form. Authors communicated that participation was voluntary and that participants could withdraw at any time. Confidentiality and anonymity were assured to all participants prior to the interviews.

## Results

Participants experienced availability, continuity, structure and good communication as important parts of a well-functioning care model. Most participants also stressed the importance of having a CM/RC who supported them, practically as well as emotionally, on their way to recovery. They experienced that people who really cared about their health and well-being had led them through their period of sick leave.

Through the inductive analysis, STC by Malterud [13], five codes were produced to synthesize the results: 1) *A feeling of being taken care of*, 2) *Collaboration within the team was perceived as supportive*, 3) *An active and sensitive listener*, 4) *Structure and planning in the PCD meeting*, 5) *The PCD meeting was supportive and provided increased understanding*.

### A feeling of being taken care of

Participants described that the PCC paid attention to their CMD at an early stage, and that this immediate feedback on their illness had felt very valuable. Participants expressed a safety in speaking to a professional with profound knowledge about their CMD, who at an early stage explained the meaning of this diagnosis to them. At first, some participants had questioned whether they really needed to be on sick leave. However, as soon as they realized the gravity of their situation, they expressed a gratitude towards the CM/RC who had convinced them.

I didn´t go home because I didn´t like my work. I went home because the PCC called me and said: “now you need to leave your job and come to us. You will have a stroke, at any minute, due to your high blood pressure”. I really didn´t get it, because I didn´t feel sick (Woman 63 years old).

Participants experienced a safety in being able to contact the CM whenever they felt the need. They had access to a direct phone number and could speak to a CM who was familiar with their situation. Most participants expressed that it was comfortable to speak with the same person, and that this enabled them to pick up from where they had left off in their last conversation. They also found a huge safety in receiving support and help from their personal CM without much delay.

Yes, this immediate feedback that was received—was the best part of the model. Because, when you feel really fragile… when you have gotten to a point that you cannot go to work any longer. To receive feedback when you feel like this is extremely valuable (Woman 62 years old).

Participants described that the model had worked as a continuous and secure foundation on their way to recovery. They also experienced a security in that all follow-ups were based on a care plan that contained scheduled meetings and medical treatments. This formal care plan was seen as an assurance that they were well taken care of and that rehabilitation plans would not run into the sand. This feeling of being taken care of made participants more relaxed and allowed them to focus on getting well.

A few participants experienced that the CM had been difficult to get in touch with or difficult to develop a personal relationship with. In these cases, participants had not understood the concept of the CM, or how they could personally benefit from this contact. These participants wished that they had received more visits at the PCC and more phone calls from the CM. Some participants experienced a lack of continuity concerning their medication and explained that they had to change doctor several times. This situation was due to the locum-tenancy situation in Swedish primary care.

### Collaboration within the team was perceived as supportive

To have a CM that was well informed and who could communicate the patient’s interests within the group was perceived as a great relief for the participants. Not having to explain their CMD over and over again to different people was highlighted as especially relieving, particularly for those who felt emotionally exhausted. Participants experienced a close collaboration among the CM, the RC, and the doctor. In particular, that the CM could communicate vital information to the doctor was seen as a crucial part of this collaboration.

I understood that the RC and the doctor communicated about my case, and that they discussed what would be the best for me, between my visits. I think this is exactly how it should work out. (Man 40 years old)

Participants appreciated that they had received practical help, in that their CM and RC contacted, for example, the doctor, the employer, or the Social Insurance Agency for them. For those participants who described their relationship to their employer as dysfunctional or who had a continuing dispute with the Social Insurance Agency, this sort of support was described as especially important.

To have one person that can help you, it can be really nice (…) When I had a dispute with the Social Insurance Agency, I asked the RC “can you please call them, because I have a lack of energy”. She answered; “yes of course”. Then she sorted out a meeting with my boss, and that was also really nice. I wouldn´t have taken that initiative myself (Woman 25 years old).

Participants had different experiences depending on whether the CM and the RC had been the same person in both roles, or if there had been two persons. Increased continuity and reduced risk for misunderstandings were brought up as potential benefits with having one person in both roles. On the other hand, participants stressed that it could be a weakness in the model, if this vital person became ill or had to leave for some reason, and the person with CMD would then end up with no one. Participants who had experienced having one person as their CM and another one as their RC stressed that this was a guarantee for a better model, since personality clashes sometimes occurred, and they had gotten along better with one of the two.

### An active and sensitive listener

Participants described their CM as sensitive to their needs and caring. Participants expressed that their needs of having someone who engaged in and listened to their situation were fulfilled. It had also been important that the CM was not a part of their inner circle of close family and friends. Participants highlighted that the CM had helped them to put their emotions into words and had provided them with various tools in order to cope with their situation. The CM and the RC had also used a pedagogical way of speaking, which participants appreciated, as they felt very tired and unfocused.

What I remember the most is the way that she spoke to me. They used an adapted language, which was easier to understand for persons with fatigue. It was therefor easy to speak with them… Yes, clear communication, I appreciated that a lot (Woman 28 years).

Participants emphasized that the CM/RC had seen and treated them as a whole person, not just as a patient with CMD. This individual treatment made them feel more valuable and less like just another patient. Overall, participants experienced that they were in safe hands and felt supported by their CM and RC during the entire period of sick leave.

### Structure and planning in the PCD meeting

Participants stressed that having the PCD meeting at the PCC had been important to them. In addition, they expressed a disappointment when this had not happened due to Covid-19. Participants emphasized that it was important to hold the meeting on neutral ground, because employers then had to leave their usual role of being in charge. Having the meeting at the PCC was also emphasized as vital for strengthening the overall impression of CMD as an actual and serious illness. Participants described that the RC had explained the impact that CMD could have on a person’s health, which had led to a discussion about possible arrangements or improvements at the workplace.

We talked about how easily I could get sick again, and the purpose (with this meeting) is for the person on sick leave to return to work, and I thought that was good, but it was also concrete examples (for how this could work out)… You can either speak at a theoretical level, or you can speak so that people will understand you, and the latter, I think she (RC) did that very well (Woman 63 years old).

Participants expressed that the RC had guided the conversation at the PCD meeting in a professional way, for example, by distributing the conversation time and allowing people who were speaking to finish their points. That the RC took a mediating role was seen as a way to avoid conflicts and to increase the chances of reaching mutual agreements during the meeting. Some participants felt well prepared for the meeting, while others stressed that they had not been told about the purpose of the meeting beforehand. Participants who were not prepared for the meeting did not feel that the meeting had been worthwhile or meaningful for them.

For many participants, the PCD meeting led to joint decisions about adjusted working conditions, such as different working hours, job assignments, or relocations. After the meeting, both parties were asked to think about how they could proceed, based on what had been decided. Some participants found it more difficult to explain what they needed, or how the workplace could be adjusted for them to go back.

No, we didn´t really come up with a solution… It was a pity, but I don´t think I was able to explain what kind of adjustments that I needed, in order to go back… it was really hard to explain. I wasn´t prepared for how to explain my situation or what to say… It was also difficult to speak about this in front of my employer (Man 51 years old).

### The PCD meeting was supportive and provided increased understanding

Participants perceived that they had been encouraged to tell their story in their own words during the PCD meeting. This was experienced as relieving, but also challenging to confront their employer with this type of information. In these situations, the RC was described as supportive and affirmative.

But, above all, I felt that I was supported. It is not just that I come and say that I feel sick, and everyone can choose to ignore me if they like… In this situation (PCD meeting) there was more substance behind it (Woman 42 years old).

In cases when the participant had a more complicated relationship with their employer, the supportive role of the RC in the meeting was described as more important to the participants. Some participants experienced that they would not have agreed to have this meeting without the RCs’ mediating role.

Participants expressed that the RC had conveyed a feeling that “this is not your fault” during the meeting, and because of that they had felt safe to speak more openly. The RCs’ ability to make the dialogue more lighthearted and less severe had been meaningful to the participants.

Several participants highlighted that the PCD meeting had provided an enhanced understanding of their employers’ pre-conditions, for example obligations and restrictions that their employers had to relate to. The PCD meeting was an eye-opener also for their employers, in that it gave them an understanding of how participants had experienced their unsustainable work situation. Participants’ overall view was that both parties could better understand one another after the PCD meeting.

It felt really good. I felt that we were on better terms afterwards. My employer and I, we know each other a little better now. I would say that we reached a better understanding of each other (Woman 39 years old).

## Discussion

This study explored participants’ experiences of their participation in the Co-Work-Care model. The aim of this model was to increase the quality of care for patients with CMD in primary care, primarily through increased collaboration between professionals working with patients and their employers through a person-centered dialogue meeting.

A main finding was that participants in general experienced that having a CM and a RC to lean on during their rehabilitation period was highly valuable. Participants expressed how their condition made them feel extremely tired and devoid of initiative. Through a close contact with their CM, they could share needs and communicate treatment preferences and other requests. In addition, the current study showed that participants valued being influential in their own care plan. Previous research showed that when patients are involved in the design of their care, patient’s motivation may increase and the forming of a partnership between the CM and the patient may be facilitated [16,17]. Assisting the patient with practical help and other issues in this vulnerable state should not be confused with the risk of disempowering the patient [18]. In our study, participants communicated that they would not have been able to manage in the early stage of their illness without support and practical help from the CM/RC. An important and sometimes difficult part of this working method lies in the CM/RC’s ability to sense where in the process the patient is, in order to provide tailored support and adequate help, without taking over too much of the individual´s own responsibilities [12,19].

Empathy, trust, and availability were highlighted as key components for a well-functioning relationship with the CM/RC. Several studies showed the importance of these components when forming an alliance or partnership between the patient and their CM [12,18,20]. Aside from these better known characteristics of well-functioning relationships with patients [21], being acknowledged as a person with individual needs was recently found to be a crucial part of the recovery process (20), which was also confirmed by the current study. In addition, participants in our study wanted to be treated as an individual, by a committed CM/RC who seemed to care for them. Taken together, all these expectations and wishes concerning how a CM/RC preferably should be place high demands on both of these roles.

Participants emphasized the importance of continuity, availability, and clear communication for experiencing a well-functioning care model. Shortcomings within these areas were associated with more negative experiences of the care process, also confirmed by previous research [22]. Furthermore, participants felt relieved when their information was communicated among the CM, RC, and the GP, so they did not have to repeat their stories several times. In line with previous research [18], the close communication between the CM and the GP made patients feel safe and well taken care of. Other previous studies confirm the increased values for patients when there is a focus on collaborative care management at the PCC [19,20,23].

Before and during the PCD meeting, structure and planning were important factors for participants to feel safe and involved in the process. Previous research showed that patients felt safer and more involved in their care, when it was developed together with the patient and when patients knew what to expect from their care plan [23,24]. Therefore, a focus on involving the patients in forming the care plan as well as preparing them for their PCD meeting seemed to be important parts of a well-functioning CWC model [24].

Participants in our study emphasized the importance of receiving support when communicating and trying to solve difficult situations with their employer in the PCD meeting. The value of supporting the patient in a situation where they often feel fragile, tired, and confused, and when the outcome of the meeting is of great importance to the patient should not be underestimated. It was confirmed that patients with CMD benefitted from emotional exposure, to move forward in their RTW process [15]. The emotional exposure could be about expressing feelings of vulnerability and self-blame or communicating experiences of lack of support from the workplace at a convergence dialogue meeting [15]. In addition, our study participants felt worried about expressing feelings in front of their employer, and support from the RC was highlighted as an important prerequisite for them to do this. Worries that were experienced could be about being judged or creating a conflict with the employer that could lead to loss of their job [6,15,25]. Our belief is that the communication at the PCD meeting needs to be respectful and honest, and the patients’ experiences need to be taken seriously.

In all, a well-structured PCD meeting, including a pedagogical framework, has the potential to promote preconditions for the RTW process among persons with CMD. In a PCD meeting it is possible to highlight work-related obstacles, so that more targeted solutions can be developed earlier in the RTW process [12]. In line with our previous argumentation, a difficulty when implementing this care model may be that a well organized and well executed PCD meeting places high demands on the individual RCs. As was indicated in the current study, RCs may need appropriate skills in communication, to maintain neutrality, but also to be able to handle conflicts that may arise during the meeting. In some meetings they need to work as a bridge between the patient and the employer, when the air is filled with unresolved conflicts. These findings indicate that the PCD meeting may need a flexible design that can be adjusted according to the state of the patient and the severity of the overall situation. For example, it may be necessary to schedule more than one meeting or to allow RCs with more extensive experience to lead meetings that are expected to be difficult.

In conclusion, the current study showed that the motivational benefits for the patients in having a RC following the RTW process and supporting them in their relationship with the employer were in general experienced as invaluable. In addition, we argue that the CM and RC need to be provided with appropriate prerequisites, so that they can live up to the role of a well-functioning CM/RC. To avoid confusion concerning how the CM and RC should work, it may be helpful to delineate the characteristics of these roles in the manual for the collaborative care model. However, as these roles may be emotionally demanding and will require a certain amount of time, the CM/RC needs to be given the right conditions in order to live up to his or her role. Thus, we argued that the CM and RC need to take the available course for how to perform in their roles [12,26]. Moreover, they also need support from and close collaboration with the GPs [27], and they need to be provided with enough time for each patient [15].

### Strengths and limitations

The structured design and thorough planning prior to the study made the intervention predictable and organized. Knowledge from the previous study about experiences of the CWC model from a CM/RC’s point of view [12] was considered when forming the interview guide, even though the concept of open-ended questions was used to let participants influence the content as much as possible.

A purposive sampling procedure was performed, which provided us with rich and complex interview data. We achieved a variation in age, work experience, and background, as well as enrollment at different PCCs and different time spans between completed intervention and time of the interview. The balance between men and women in this study is similar to the real distribution of men and women with a CMD diagnosis. However, we did not succeed in including foreign-born participants, which is a limitation of this study.

Members of the research team had different areas of expertise, which made the analysis nuanced, and a deeper interpretation of the results was obtained through discussions. All authors were involved in the analysis and in these discussions, contributing to more credible and trustworthy results.

It is difficult to speculate about how the covid-19 situation and especially the telephone interviews influenced the outcome of the study. Known weaknesses related to phone interviews are the lack of facial expressions and other non-verbal signals that may lead to a weaker personal engagement [28]. However, several participants mentioned that they felt more comfortable about reliving certain thoughts over the phone and more relaxed to disclose sensitive information. Further, these arguments were strengthened by the fact that all participants declined to be part of a video meeting online. Participants also preferred phone interviews over face-to-face interviews, due to tiredness related to their condition. The overall conclusion was therefore that the format of the interviews might have suited participants with CMD rather well.

## Conclusions

All in all, participants considered the CWC model and especially the close collaborative contact with the CM/RC as highly valuable. Participants perceived their relationship with the CM/RC as trusting, and a majority emphasized that this relationship had played a crucial role when struggling through a very difficult period in life. With regard to the PCD meeting, participants valued a well-structured meeting that included initial planning and a thorough communication among all parties. Participants appreciated the active role that they were encouraged to take during the meeting. Further, it seemed that this active engagement contributed to empower participants in their RTW process.

The PCD meeting serves as a bridge between health care and work life that helps to contribute to a work-orientated focus on rehabilitation for persons with CMD. A properly elaborated and well- structured CWC model that includes a PCD meeting has considerable potential to contribute to shortened periods of sick leave and a more sustainable RTW for persons with CMD.

## Supporting information

S1 File Interview guide in Swedish. (PDF)

S2 File Interview guide in English. (PDF)
