# Supplementary material for: Experiences of patients with common mental disorders concerning team-based primary care and a person-centered dialogue meeting: An intervention to promote return to work
Source: PLoS One. 2022 Jul 8;17(7):e0271180. doi: 10.1371/journal.pone.0271180 (PMC9269955; doi:10.1371/journal.pone.0271180)
Supplement: S1 File — (PDF) [file pone.0271180.s001.pdf]

Intervjuguide – Co-Work-Care

Patienten informeras om projektet och projektets syfte (Se Etikprövning och det informerade samtycket)

Presentation av personer som är med under intervjun, samt datum och klockslag.

Inledning

Vi tänker inledningsvis fråga dig om den modell som användes i studien. Den här modellen innebar ett ökat samarbete mellan vårdsamordnare, rehabkordinator och läkare, (samt ett samtal med arbetsgivaren som vi återkommer till).

Hur upplever du att kontakten med vårdsamordnare har fungerat?

Vilka är dina upplevelser av stödet du fått från vårdsamordnaren?

Hur upplever du att kontakten med rehabkoordinator har fungerat?

Vilka är dina upplevelser av stödet du fått från rehabkoordinatorn?

Hur tycker du att kontakten med läkaren har fungerat?

Vilka är dina upplevelser av stödet du fick från läkaren?

Hur upplever du att samarbetet mellan RK, VS och läkaren har fungerat avseende din situation?

Tyckte du att vården i ditt fall var tillräckligt tillgänglig? (t.ex. hade du önskat mer kontakt med VS, RK eller läkare?)

Var det något som du tyckte var särskilt bra med den här modellen – där VS, RK och läkare ingick?

Fanns det saker som du tyckte fungerade sämre med modellen?

Konvergenssamtalet

Nu tänkte vi fråga dig lite mer om hur du upplevde det här samtalet med arbetsgivare och rehabkoordinator, det s.k. konvergenssamtalet.

Hur upplevde du samtalet med arbetsgivaren där rehabkoordinator var samtalsledare?

På vilket sätt fick du möjlighet att berätta om din situation?

Gav samtalet dig någon ny kunskap om arbetsplatsens förutsättningar för din arbetsåtergång? (alltså hur du skulle kunna komma åter i arbete)

Den här modellen innebar framförallt ett ökat samarbete mellan RK, VS och läkaren. Tyckte du att detta märktes i hur samtalet blev? (T.ex. verkade det som att RK var insatt i ditt fall?)

Var det något du tyckte var särskilt bra med samtalet?

Är det något du skulle velat ändra på?

Hur upplevde du avslutningen av samtalet? (ev. Hur kändes det efter mötet?)

Avslutningsfråga

Av det som du nu berättat om – vad tycker du har varit särskilt viktigt i det strukturerade samarbetet och samtalet med arbetsgivaren? Vilka delar vill du att vi behåller? Är det något som du vill ändra på? Är det något ytterligare som du gärna vill förmedla?

Hjälpfrågor - följdfrågor

- Hur tänker du då?

- Skulle du vilja ge ett exempel på en sådan situation?

- Kan du berätta mer om det?

-Jag förstår inte riktigt, hur hänger det ihop? (att motivera uttalanden)

-Hur kom du fram till det?

-Hur upplevde du det?

-Hur viktigt är detta för dig?

-Påminna om ämnet – vi är intresserade av så många olika synpunkter och olika upplevelser som möjligt!
