# Supplementary material for: Experiences of patients with common mental disorders concerning team-based primary care and a person-centered dialogue meeting: An intervention to promote return to work
Source: PLoS One. 2022 Jul 8;17(7):e0271180. doi: 10.1371/journal.pone.0271180 (PMC9269955; doi:10.1371/journal.pone.0271180)
Supplement: S2 File — (PDF) [file pone.0271180.s002.pdf]

Interview Guide – Co-Work-Care

The patient is informed about the study and the study's purpose. (See Ethical Approval and informed consent.)
The persons who are present during the interview are presented; date and time are noted.

Introduction

We would first like to ask you about the model that was used in the study. This model involves an increased cooperation among the care managers, the rehabilitation coordinators, and the doctors (as well as a conversation with the employer, which we will return to.)

How did you experience the contact with the care manager?

How did you experience the support that you received from the care manager?

How did you experience the contact with the rehabilitation coordinator?

How did you experience the support that you received from the rehabilitation coordinator?

How did you experience the contact with the doctor?

How did you experience the support that you received from the doctor?

How did you experience the cooperation among the rehabilitation coordinator, the care manager, and the doctor with regard to your situation?

Did you think that you had sufficient access to care? (For example, would you have liked to have had more contact with the rehabilitation coordinator, care manager, or doctor?)

Was there anything that was especially good with this particular model, which included a care manager, rehabilitation counselor, and doctor?

Were there any aspects of the model which you thought did not work as well?

Person-centered Dialogue Meeting

Now we would like to ask you a bit more about your experiences with regard to the dialogue meeting with the employer and the rehabilitation coordinator.

How did you experience the meeting with the employer during which the rehabilitation coordinator acted as the leader of the dialogue?

In what way did you get an opportunity to describe your situation?

Did the meeting provide any new knowledge about workplace prerequisites for your return to work? (That is, how might you be able to go back to working there again?)
This particular model involves above all an increased cooperation among the rehabilitation coordinator, the care manager, and the doctor. Was this noticeable during the course of the dialogue meeting? (For example, did it seem as if the rehabilitation coordinator was familiar with your case?)

Was there anything that you thought was especially good about the dialogue meeting?

Is there anything that you would have liked to change?

How did you experience the conclusion of the meeting? (How did you feel after the meeting?)

Final question

With regard to what you have told us about – what do you think is especially important in the structured cooperation and the dialogue meeting with the employer? Which aspects would you like us to keep? Is there anything that you would like to change? Do you have any further comments that you would like to provide?

Help questions – probes

- What did you think about that?
- Could you give an example of such a situation?
- Can you tell us more about that?
- I do not really understand. How does that hang together? (Can you tell me why you say this?)
- How did you come to this realization?
- How did you experience that?
- How important is that for you?
- Remind the person about the topic – we are interested in getting as many different viewpoints and experiences as possible.
